# Robust Approaches for the Production of Active Ingredient and Drug Product for Human Phage Therapy

**DOI:** 10.3389/fmicb.2019.02289

**Published:** 2019-10-08

**Authors:** Michele Mutti, Lorenzo Corsini

**Affiliations:** PhagoMed Biopharma GmbH, Vienna, Austria

**Keywords:** phage therapy, quality by design, PhagoMed, bacteriophages, antibiotic resistance, phage purification

## Abstract

To be successful, academic and commercial efforts to reintroduce phage therapy must ensure that only safe and efficacious products are used to treat patients. This raises a number of manufacturing, formulation, and delivery challenges. Since phages are biologics, robust manufacturing processes will be crucial to avoid unwanted variability in each step of the process. The quality standards themselves need to be developed, as patients are currently being treated with phages produced under quality standards ranging from cGMP for clinical trials in EMA and FDA regulated environments to no standards at all in some last resort treatments. In this short review, we will systematically review the literature covering technical issues and approaches to increase robustness at every step of the production process: the identity of the phage and bacterial production strains, the fermentation process and purification, the formulation of the drug product, the quality controls and the documentation standards themselves. We conclude that it is possible to control cost at the same time, which is critical to re-introduce phage therapy to western medicine.

## Introduction

The emergence of antimicrobial resistances (AMR) stimulated the research and development of alternatives to antibiotics, including antibodies, peptides, endolysins, and bacteriophages. Bacteriophages have very desirable characteristics, such as their ability to propagate at the site of infection and low toxicity (Pirnay et al., 2015). However, phages also have drawbacks inherent with their nature as replicating viruses, such as their limited host range, and the high rate of phage-resistant mutants. These drawbacks complicate manufacturing through the need for phage cocktails. The stability of these cocktails can vary, an effect which recently contributed to the failure of a clinical trial (Jault et al., 2019). The need for propagation on bacteria results in challenges in the purification, a drawback shared with some other biopharmaceuticals. The formulation of phages in a drug product has been characterized only for few applications (Brown et al., 2016, 2017a,b, 2018; Merabishvili et al., 2017). In order to achieve a broader application in humans, phage therapy must comply with the strict regulations for pharmaceutical products, which have not been developed with phages in mind, so that the application of these regulations poses challenges of its own (Pirnay et al., 2018b). In this article, we will systematically review literature on solutions for technical challenges associated with the production of phages under the international guidelines of Good Manufacturing Practice (GMP).

## Legal Framework

In Europe, the directive 2001/83/EC provides the framework for regulations on pharmaceuticals and medicinal products, and clinical trials are regulated by the directive 2001/20/EC and regulation No 536/2014. These were implemented as national legislation in all member states of the European Union. In the USA, Title 21 of the Code of Federal Regulations (21 CFR) regulates current Good Manufacturing Practice (cGMP) and clinical trials. To define more detailed requirements, the European, US, Japanese, and other regulatory bodies adopted common guidelines to harmonize the development and production of pharmaceuticals, through the International Conference on Harmonization (ICH). For the scope of this review, the most important ICH guidelines are ICH Q7 (n.d.), which defines cGMP as well as underlying guidelines ICH Q5B (n.d.), ICH Q5D (n.d.) (Biotechnological Products), Q8(R2) (Pharmaceutical Development), ICH Q9 (n.d.) (Quality Risk management), and ICH Q10 (n.d.) (Quality System).

So far, every modern controlled trial of a fixed phage product that was designed to establish efficacy failed to demonstrate it (Sarker et al., 2016; Jault et al., 2019). However, literature suggests that for specific indications, phage therapy could prove to be even superior to the current standard of care (Fish et al., 2018). To establish phage therapy as a first-line treatment in these cases, marketing authorization as a pharmaceutical and production in GMP will likely be required. Recently, registration of some phages as magistral ingredients, for which GMP and the above guidelines are currently not applicable, was implemented in Belgium, and these were applied to individual patients (Pirnay et al., 2018a; Djebara et al., 2019). It is still open whether this approach can achieve wide-spread use internationally also in cases where a scientifically validated standard of care can be expected to be effective (e.g., where bacteria are susceptible to antibiotics), meaning outside of the last resort cases of Article 37 of the Helsinki Declaration. If the magisterial route will be applied on a larger scale and many different parties start producing phage products for patients, we expect that GMP or something close will be demanded by the authorities to safeguard patient safety. Therefore, we will focus this mini-review on phages produced under GMP.

## Quality Standards

Quality by Design (QbD) is regarded as the most effective concept for establishing a robust process for the manufacture of drug products (DP) that are consistently effective and safe (ICH Q8(R2), n.d.; Debevec et al., 2018). The development of a manufacturing process following QbD starts by identifying the Quality Target Product Profile (QTPP), i.e., the intended clinical setting, administration route, dosage, container system, and storage of the DP (Yu et al., 2014). The QTPP delineates the Critical Quality Attributes (CQAs), the biological, chemical, microbiological, and physical characteristics that the product must have. For bacteriophages-based DP, the CQAs would typically include identity, absence of contaminating phages, titers of each phage present in the cocktail, maximum level of bacterial toxins and other contaminants, pH, sterility, and shelf life (Pirnay et al., 2015). The systematic development under QbD follows the concept of the Design Space (DS), a “multidimensional combination and interaction of input variables and process parameters that have been demonstrated to provide assurance of quality.” The dimensionality of the DS is determined by the Critical Process Parameters (CPPs), the understanding of which allows to consistently meet the specifications of the CQAs, throughout the product life cycle and even after changes in external factors. In contrast, a rigid definition of the production process, relying on the knowledge of only few variables, can lead to failures in product quality when changes have unforeseen effects (Debevec et al., 2018).

## Selected Elements of the Design Space for Phage-Based Pharmaceuticals

### Identity of the Phage and the Bacterial Production Strains

Only phages that do not contain genetic elements encoding for lysogeny, antimicrobial resistance, and virulence factors are currently considered suited for phage therapy (Pirnay et al., 2015), which can be confirmed by sequencing and transduction tests. A potential concern is also contamination with phages not intended as active ingredient (AI), which can interfere with the fermentation process or even overgrow the AI phages. The contaminations can arise from the raw materials (water or nutrients), from cross-contaminations with other phages used in the same production facilities, or from induction of prophages from the host bacterial strain (Jones et al., 2000; Łoś et al., 2004; Samson and Moineau, 2013). Therefore, the risk of contaminating phages must first be assessed during the process design, and the results must be used to design appropriate quality controls (QCs). The DS must include strategies to avoid contamination with non-intended phages. Also the host strain used for production strongly influences the phage purification process, and should be thoroughly characterized as part of the process development.

### Use of Genetically Modified Bacterial Strains for Phage Production

While the pathway for regulatory approval of genetically modified (GM) phages remains largely unexplored, phages genetically “cured” of lysogeny were successfully used to treat a patient (Dedrick et al., 2019). The use of GM bacterial hosts might be easier to adopt, since they are already approved and in use in other sectors (Lee et al., 2008; Luo et al., 2018; Zhang et al., 2018), and there are detailed guidelines for their use (ICH Q5; ICH Q6A (n.d.), ICH Q6B (n.d.); ICH S6 (R1) (n.d.)). Genetic modifications of the bacterial host can improve phage titers, ease the purification process (Ceglarek et al., 2013), or “detoxify” the host, for example by removing prophages (Bae et al., 2006; Souvignier et al., 2015; Euler et al., 2016).

### Fermentation Process

Generally, fermentation process parameters are mainly optimized to improve the phage titer, to increase the phage/impurity ratio, and to reduce the overall cost of production. Bacterial and phage inocula, type of nutrients, agitation/oxygenation, and temperature strongly influence the phage titer (Ali et al., 2019). In addition, an increase of the pH to 8.0 significantly decreased the expression of enterotoxin in *S. aureus* fermentations (Metzger et al., 1973). Industrial-scale fermentation of phages would typically be carried out in bioreactors, in fed-batch, continuous (Mancuso et al., 2018), or semi-batch (Sauvageau and Cooper, 2010) mode. Of these, the latter has the distinct advantage to allow for a continuous production mode while avoiding co-evolution of phages and bacteria (Mizoguchi et al., 2003; Jurač et al., 2019; Yuan et al., 2019).

### Purification of Phages

The purification process must be designed to consistently achieve the CQA specifications. Among the substances which most strongly influence the safety of the DP are endotoxins, which can also be introduced through raw materials and water. Bacterial toxins such as enterotoxins, alpha-toxin, and several enzymes are also undesired (Otto, 2014). Microbial DNA of various species, other bacterial compounds, and phage dsRNA can induce inflammation (Hemmi et al., 2000; Dalpke et al., 2006; Garantziotis et al., 2007; Sweere et al., 2019). Some phages degrade the DNA of the host, however, not consistently to a safe level (Kutter et al., 2018). Proteases should be removed as they could negatively influence the shelf life of the DP.

Depending on the QTTP, the purification has to occur in multiple steps. The sterile filtered lysate can be pre-treated with enzymes, for example, to ease the downstream processes and remove contaminants (Kalyanpur, 2002). For purification, CsCl gradient centrifugation is routinely used by many academic labs, however, it suffers from low separation power compared to other methods and low scalability (Merten et al., 2005). Ultrafiltration is a highly scalable alternative and very effective in separating phages from smaller impurities (Jungbauer, 2013). However, separation of phages from endotoxin is more challenging, since it forms micelles that are of similar size or even larger than phages (Petsch, 2000). Thus, especially for Gram-negative phage lysates, additional purification steps like different types of ion-exchange chromatography (Boratyński et al., 2004; Kramberger et al., 2015), affinity chromatography (Ceglarek et al., 2013), or solvent extraction (Szermer-Olearnik and Boratyński, 2015) have been proposed to remove endotoxin and other residual contaminants, and the best choice may vary by phage (Van Belleghem et al., 2017).

### Formulation for Phage-Based Products Including Stability

For phages, many formulations ranging from sterile liquids to non-sterile oral liquids, oral solids, semisolids, and patches have been described, depending on the application (Malik et al., 2017). Despite these examples, literature data regarding formulation for phage-based products is scarce. Studies on formulations used for Adeno-associated virus vectors (AAV), which are somewhat related to bacteriophages from a biochemical perspective, can be considered as a reference (Rodrigues et al., 2019). A complete review of formulations is beyond the scope of this review; therefore, we will focus on particularly relevant aspects. A typical challenge with liquid formulations is the stability of the phages. This has contributed to the failing of the PhagoBurn trial, where the instability was discovered only during the trial (Jault et al., 2019). Low stability/decreasing potency of viruses can be the result of aggregation, adsorption to the primary container, chemical degradation, or oxidation (Rodrigues et al., 2019). Aggregation is induced mainly by electrostatic interactions, and can be prevented with charged excipients, certain non-ionic surfactants, or pH optimization. Oxidation can be reduced by anti-oxidants (Rodrigues et al., 2019). In fact, AAV-DP typically contains buffer, antioxidants, surfactants tonicity agents, and cryoprotectants if the DP is intended for storage under frozen conditions (Rodrigues et al., 2019). To define the right balance of excipients experimentally, QbD principles require a significant effort in testing the stability of the AI (all phages in case of a cocktail) formulated as the final DP, inside the primary container.

## Quality Controls

QCs ensure that the DP fulfills the specifications of the CQAs regarding the AI, the formulation and primary container, as derived from the QTTP. The guidelines EMEA/CHMP/EWP/192217/2009 Rev. (n.d.) 1 and ICH Q2 (R1) (n.d.) define the design and validation of analytical methods, and the ICH Q4 (n.d.) and ICH Q3 (n.d.) series for guidelines define specific tests. In the following, we will review the types of QCs, which are expected to be applicable to most phage products.

### Identity

As described above, the identity of each phage as an AI is a CQA, with regards to the specific genomic sequence of the phage. The identity of each phage in the master seed bank needs to be demonstrated by NGS (Pirnay et al., 2015). The maximum acceptable level of genomic divergence between the master seed lot and the phage population in the propagated DP is not defined in guidelines and therefore needs to be aligned with the authorities case by case. While random mutations during propagation are unavoidable, they need to be limited as much as possible through the design of the process, and the functional properties need to be tested with validated QCs, as even individual SNPs can lead to significant phenotypic changes (Botka et al., 2019). Metagenomics has been proposed as QC for some vaccines (Neverov and Chumakov, 2010; Cliquet et al., 2015; Höper et al., 2015; WHO Technical Report Series, 2018), and has also been used to evaluate the composition of commercial phage products (McCallin et al., 2013; Villarroel et al., 2017), or for the detection of phages and bacteria in fermentation processes (Sturino and Klaenhammer, 2006; Samson and Moineau, 2013; De Filippis et al., 2017). Depending on the risk, QCs for the identity of the AI-phage and phage contaminants might be required for each batch of DP. However, in this case, instead of NGS, a highly discriminating PCR-based genotyping technique might be sufficient (Pirnay et al., 2015).

### Titer

The titer of each individual phage in the DP is typically assessed by the double agar layer method (DAL). An alternative to DAL are time-kill assays, where the kinetics of the phage-induced lysis is assessed by measuring the optical density (Xie et al., 2018; Rajnovic et al., 2019; Storms et al., 2019). Other methods, such as qPCR and ELISA, can be used to determine the phage titer, but they rely on the quantification of single components, while the DAL and time-kill assays determine the actual functional virions (Pirnay et al., 2015).

### General Purity

For biopharmaceuticals, the purity and correct composition of the DP is typically assessed by high performance liquid chromatography, if required coupled with mass spectrometry (Rouse et al., 2017). These methods can also be used to identify the phage capsid proteins, toxins, or other bacterial proteins (Rodrigues et al., 2019). Due to the safety risk posed by the production in pathogenic bacteria, CQAs need to specify maximum levels for specific contaminants like endotoxin, enterotoxins, or bacterial DNA, which typically have to be tested with specific, appropriate methods.

### Endotoxin

Given the importance of the endotoxin testing, several *in vitro* methods have been developed: gel-clot, turbidimetric, and chromogenic methods. Among the chromogenic methods the Limulus Amebocyte Lysate (LAL) assay is the most frequently used (Abate et al., 2017). When the LAL assay is not applicable, e.g., due to the masking effect, a reporter cell line can be used (Schwarz et al., 2017).

### Contamination by Toxic Bacterial Proteins

Several commercial assays can be used to detect toxic bacterial proteins, including ELISAs for enterotoxins, or assays based on reporter cell lines.

### Nucleic Acid Contaminants

Depending on the extent that the phage already degrades bacterial DNA, QCs might be required to demonstrate that the nucleic acid concentration in the DP meets the specifications. The presence and concentration of residual nucleic acids can be tested by qPCR or by using reporter cell lines.

### Other Quality Controls

The current standard to check sterility or microbial contaminations of pharmaceuticals should be applied to phage-based pharmaceuticals as well (Shintani, 2016). Other specifications, which might need to be tested include pH, osmolarity, and visual appearance (Pirnay et al., 2018b).

## Quality System

The requirements to a quality system in pharmaceutical manufacturing are described in the guideline ICH Q10. ICH Q10 adds to Q8 (Pharmaceutical Development) and Q9 (risk management) by defining the requirements to ensure consistent product quality and thereby securing patient safety and drug efficacy. The quality system described in ICH Q10 consists of four elements: (1) a process performance and product quality monitoring system, (2) a corrective and preventative action (CAPA) system, (3) a change management system, and (4) a management review process. The guideline defines that the most senior leadership level of the company responsible for the process is ultimately accountable for product quality, so that management has the responsibility to own and operate the quality system. Importantly, the guideline also clarifies that these elements should be applied in a manner that is proportionate and appropriate for the life cycle stage the product is in.

## Existing Experience in Good Manufacturing Practice Production of Phage Cocktails

To the best of our knowledge, robust phage production processes designed according to the QbD principles are not reported. Only one study describes a quality controlled small-scale production process of a phage cocktail intended for human use, which however was not designed under GMP (Merabishvili et al., 2009). There are several reports of clinical trials where GMP produced cocktails of phages against *P. aeruginosa*, *S. aureus*, and *E. coli* were used (Wright et al., 2009; Rhoads et al., 2014; Sarker et al., 2016; Jault et al., 2019, NCT03395769, NCT03395743) or are being used (NCT02664740, NCT03808103). While these reports do not disclose details of the DS for the respective cocktails, they demonstrate that several organizations succeeded in getting clinical trials with GMP phage cocktails approved by regulatory bodies. Furthermore, all these cases and trials demonstrated excellent safety profiles, and no adverse events have been reported.

## A Robust Process vs. Low-Cost Production: Is it Really Either Or?

In general, developing a robust process according to the QbD principles will increase the cost of development (Schmitt, 2011). However, QbD will also lead to lower production cost, by reducing the risk of rejected batches, the cost of documenting process deviations, managing CAPAs, and the registration cost for process changes. The emerging phage industry will need to find the right balance for these opposing influences. Moreover, also the regulators will influence production cost, for example through the risk-based decision on the exact purity specifications. This will determine the number of purification steps, the level of process control required to consistently achieve these specifications, and the number and type of QCs, which will have to be conducted and reviewed. In fact, some reports show that adverse events were also not observed where only the endotoxin was removed from the raw lysate with Endotrap columns (Merabishvili et al., 2009; Rose et al., 2014), indicating that, at least in these cases, this process was sufficient for patient safety. Ultimately, a robust process helps ensure that the product is consistently safe and effective, and the investment needed to develop it will have to be commensurate to the intended patient population and route of administration.

## Conclusions

The ultimate goal for organizations developing phage therapy should be the wide-spread use of safe and efficacious products for the benefit of patients, even as first-line treatment. Comparing phage-based pharmaceuticals against anything less than the highest standards set by regulators would invite doubts on their effectiveness and safety. This review argues that robust production processes should be feasible for the most important aspects of production of phages. Demonstrating that phage-based drugs are effective and safe also under EMA or FDA standards is the “next level challenge” for the emerging industry. We expect that once this challenge has been overcome for first proof-of-concept examples, many more phage-based pharmaceuticals will reach the market and phages will finally reclaim their place in global medicine.

## Author Contributions

Both authors listed have made a substantial, direct and intellectual contribution to the work, and approved it for publication.

### Conflict of Interest

LC and MM were employed by the company PhagoMed Biopharma GmbH. LC owns shares in PhagoMed Biopharma GmbH.

## References

[ref1] Abate W. Sattar A. A. Liu J. Conway M. E. Jackson S. K. (2017). Evaluation of recombinant factor C assay for the detection of divergent lipopolysaccharide structural species and comparison with limulus amebocyte lysate-based assays and a human monocyte activity assay. J. Med. Microbiol. 66, 888–897. 10.1099/jmm.0.000510, PMID: 28693666

[ref2] Ali J. Rafiq Q. Ratcliffe E. (2019). A scaled-down model for the translation of bacteriophage culture to manufacturing scale. Biotechnol. Bioeng. 116, 972–984. 10.1002/bit.26911 30593659

[ref3] Bae T. Baba T. Hiramatsu K. Schneewind O. (2006). Prophages of *Staphylococcus aureus* Newman and their contribution to virulence. Mol. Microbiol. 62, 1035–1047. 10.1111/j.1365-2958.2006.05441.x, PMID: 17078814

[ref4] Boratyński J. Syper D. Weber-Dąbrowska B. Łusiak-Szelachowska M. Poźniak G. Górski A. (2004). Short communication preparation of endotoxin-free bacteriophages. Cell. Mol. Biol. Lett. 9, 253–259. 15213806

[ref5] Botka T. Pantůček R. Mašlaňová I. Benešík M. Petráš P. Růžičková V. (2019). Lytic and genomic properties of spontaneous host-range Kayvirus mutants prove their suitability for upgrading phage therapeutics against staphylococci. Sci. Rep. 9:5475. 10.1038/s41598-019-41868-w 30940900 PMC6445280

[ref6] Brown T. L. Petrovski S. Chan H. T. Angove M. J. Tucci J. (2018). Semi-solid and solid dosage forms for the delivery of phage therapy to epithelia. Pharmaceuticals 11:26. 10.3390/ph11010026, PMID: 29495355 PMC5874722

[ref7] Brown T. L. Petrovski S. Dyson Z. A. Seviour R. Tucci J. (2016). The formulation of bacteriophage in a semi solid preparation for control of *Propionibacterium acnes* growth. PLoS One 11:e0151184. 10.1371/journal.pone.0151184, PMID: 26964063 PMC4786141

[ref8] Brown T. L. Petrovski S. Hoyle D. Chan H. T. Lock P. Tucci J. (2017a). Characterization and formulation into solid dosage forms of a novel bacteriophage lytic against *Klebsiella oxytoca*. PLoS One 12:e0183510. 10.1371/journal.pone.0183510 28817689 PMC5560551

[ref9] Brown T. L. Thomas T. Odgers J. Petrovski S. Spark M. J. Tucci J. (2017b). Bacteriophage formulated into a range of semisolid and solid dosage forms maintain lytic capacity against isolated cutaneous and opportunistic oral bacteria. J. Pharm. Pharmacol. 69, 244–253. 10.1111/jphp.12673 28033675

[ref10] Ceglarek I. Piotrowicz A. Lecion D. Miernikiewicz P. Owczarek B. Hodyra K.. (2013). A novel approach for separating bacteriophages from other bacteriophages using affinity chromatography and phage display. Sci. Rep. 3:3220. 10.1038/srep03220, PMID: 24225840 PMC3827602

[ref11] Cliquet F. Picard-Meyer E. Mojzis M. Dirbakova Z. Muizniece Z. Jaceviciene I.. (2015). In-depth characterization of live vaccines used in Europe for oral rabies vaccination of wildlife. PLoS One 10:e0141537. 10.1371/journal.pone.0141537, PMID: 26509266 PMC4625003

[ref12] Dalpke A. Frank J. Peter M. Heeg K. (2006). Activation of toll-like receptor 9 by DNA from different bacterial species. Infect. Immun. 74, 940–946. 10.1128/IAI.74.2.940-946.2006, PMID: 16428738 PMC1360326

[ref13] De Filippis F. Parente E. Ercolini D. (2017). Metagenomics insights into food fermentations. Microb. Biotechnol. 10, 91–102. 10.1111/1751-7915.12421, PMID: 27709807 PMC5270737

[ref14] Debevec V. Srčič S. Horvat M. (2018). Scientific, statistical, practical, and regulatory considerations in design space development. Drug Dev. Ind. Pharm. 44, 349–364. 10.1080/03639045.2017.1409755 29200316

[ref15] Dedrick R. M. Guerrero-Bustamante C. A. Garlena R. A. Russell D. A. Ford K. Harris K.. (2019). Engineered bacteriophages for treatment of a patient with a disseminated drug-resistant *Mycobacterium abscessus*. Nat. Med. 25, 730–733. 10.1038/s41591-019-0437-z, PMID: 31068712 PMC6557439

[ref16] Djebara S. Maussen C. De Vos D. Merabishvili M. Damanet B. Pang K.. (2019). Processing phage therapy requests in a Brussels military hospital: lessons identified. Viruses 11:265. 10.3390/v11030265, PMID: 30884879 PMC6466067

[ref17] EMEA/CHMP/EWP/192217/2009 Rev (n.d.). 1 Bioanalytical method validation. Available at: https://www.ema.europa.eu/en/bioanalytical-method-validation (Accessed February 1, 2012).

[ref18] Euler C. W. Juncosa B. Ryan P. A. Deutsch D. R. McShan W. M. Fischetti V. A. (2016). Targeted curing of all lysogenic bacteriophage from *Streptococcus pyogenes* using a novel counter-selection technique. PLoS One 11:e0146408. 10.1371/journal.pone.0146408 26756207 PMC4710455

[ref19] Fish R. Kutter E. Wheat G. Blasdel B. Kutateladze M. Kuhl S. (2018). Compassionate use of bacteriophage therapy for foot ulcer treatment as an effective step for moving toward clinical trials. Methods Mol. Biol. 1693, 159–170. 10.1007/978-1-4939-7395-8_14 29119440

[ref20] Garantziotis S. Hollingsworth J. W. Zaas A. K. Schwartz D. A. (2007). The effect of toll-like receptors and toll-like receptor genetics in human disease. Annu. Rev. Med. 59, 343–359. 10.1146/annurev.med.59.061206.112455 17845139

[ref21] Hemmi H. Takeuchi O. Kawai T. Kaisho T. Sato S. Sanjo H.. (2000). A toll-like receptor recognizes bacterial DNA. Nature 408, 740–745. 10.1038/35047123, PMID: 11130078

[ref22] Höper D. Freuling C. M. Müller T. Hanke D. von Messling V. Duchow K. (2015). High definition viral vaccine strain identity and stability testing using full-genome population data – the next generation of vaccine quality control. Vaccine 33, 5829–5837. 10.1016/j.vaccine.2015.08.091 26387431

[ref23] ICH Q10 (n.d.). Pharmaceutical Quality System. Available at: https://www.ema.europa.eu/en/ich-q10-pharmaceutical-quality-system (Accessed June 1, 2008).

[ref24] ICH Q2 (R1) (n.d.). Validation of Analytical Procedures: Text and Methodology. Available at: https://www.ema.europa.eu/en/ich-q2-r1-validation-analytical-procedures-text-methodology (Accessed June 1, 1997).

[ref25] ICH Q3 (n.d.). Impurities. Available at: https://www.ema.europa.eu/en/human-regulatory/research-development/scientific-guidelines/ich/ich-quality (Accessed August 1, 2002).

[ref26] ICH Q4 (n.d.). Regulatory Acceptance. Available at: https://www.ema.europa.eu/en/human-regulatory/research-development/scientific-guidelines/ich/ich-quality (Accessed June 1, 2008).

[ref27] ICH Q5B (n.d.). Analysis of the Expression Construct in Cell Lines Used for Production of rDNA-Derived Protein Products. Available at: https://www.ema.europa.eu/en/ich-q5b-analysis-expression-construct-cell-lines-used-production-rdna-derived-protein-products (Accessed June 1, 1996).

[ref28] ICH Q5D (n.d.). Derivation and Characterisation of Cell Substrates Used for Production of Biotechnological/Biological Products. Available at: https://www.ema.europa.eu/en/ich-q5d-derivation-characterisation-cell-substrates-used-production-biotechnologicalbiological (Accessed June 1, 1996).

[ref29] ICH Q6A (n.d.). Test Procedures and Acceptance Criteria for New Drug Substances and New Drug Products: Chemical Substances. Available at: https://www.ema.europa.eu/en/ich-q6a-specifications-test-procedures-acceptance-criteria-new-drug-substances-new-drug-products (Accessed May 1, 2000). 12356095

[ref30] ICH Q6B (n.d.). Test Procedures and Acceptance Criteria for Biotechnological/Biological Products. Available at: https://www.ema.europa.eu/en/ich-q6b-specifications-test-procedures-acceptance-criteria-biotechnologicalbiological-products (Accessed May 1, 2000).

[ref31] ICH Q7 (n.d.). Good Manufacturing Practice for Active Pharmaceutical Ingredients. Available at: https://www.ema.europa.eu/en/ich-q7-good-manufacturing-practice-active-pharmaceutical-ingredients (Accessed November 1, 2000).

[ref32] ICH Q8(R2) (n.d.). Pharmaceutical Development. Available at: https://www.ema.europa.eu/en/ich-q8-r2-pharmaceutical-development

[ref33] ICH Q9 (n.d.). Quality Risk Management. Available at: https://www.ema.europa.eu/en/ich-q9-quality-risk-management (Accessed May 28, 2014).

[ref34] ICH S6 (R1) (n.d.). Preclinical Safety Evaluation of Biotechnology-Derived Pharmaceuticals. Available at: https://www.ema.europa.eu/en/ich-s6-r1-preclinical-safety-evaluation-biotechnology-derived-pharmaceuticals (Accessed March 1, 1998). 10.1038/nrd822 12119749

[ref35] Jault P. Leclerc T. Jennes S. Pirnay J. P. Que Y.-A. Resch G. (2019). Efficacy and tolerability of a cocktail of bacteriophages to treat burn wounds infected by *Pseudomonas aeruginosa* (PhagoBurn): a randomised, controlled, double-blind phase 1/2 trial. Lancet Infect. Dis. 19, 2–3. 10.1016/S1473-3099(18)30482-1 30292481

[ref37] Jones D. T. Shirley M. Wu X. Keis S. (2000). Bacteriophage infections in the industrial acetone butanol (AB) fermentation process. J. Mol. Microbiol. Biotechnol. 2, 21–26. PMID: 10937483

[ref38] Jungbauer A. (2013). Continuous downstream processing of biopharmaceuticals. Trends Biotechnol. 31, 479–492. 10.1016/j.tibtech.2013.05.011, PMID: 23849674

[ref39] Jurač K. Nabergoj D. Podgornik A. (2019). Bacteriophage production processes. Appl. Microbiol. Biotechnol. 103, 685–694. 10.1007/s00253-018-9527-y, PMID: 30474729

[ref40] Kalyanpur M. (2002). Downstream processing in the biotechnology industry. Mol. Biotechnol. 22, 087–098. 10.1385/MB:22:1:087 12353915

[ref41] Kramberger P. Urbas L. Štrancar A. (2015). Downstream processing and chromatography based analytical methods for production of vaccines, gene therapy vectors, and bacteriophages. Hum. Vaccin. Immunother. 11, 1010–1021. 10.1080/21645515.2015.1009817, PMID: 25751122 PMC4514237

[ref42] Kutter E. Bryan D. Ray G. Brewster E. Blasdel B. Guttman B. (2018). From host to phage metabolism: hot tales of phage T4’s takeover of *E. coli*. Viruses 10:387. 10.3390/v10070387, PMID: 30037085 PMC6071114

[ref43] Lee S. K. Chou H. Ham T. S. Lee T. S. Keasling J. D. (2008). Metabolic engineering of microorganisms for biofuels production: from bugs to synthetic biology to fuels. Curr. Opin. Biotechnol. 19, 556–563. 10.1016/j.copbio.2008.10.014, PMID: 18996194

[ref44] Łoś M. Czyz A. Sell E. Wegrzyn A. Neubauer P. Wegrzyn G. (2004). Bacteriophage contamination: is there a simple method to reduce its deleterious effects in laboratory cultures and biotechnological factories? J. Appl. Genet. 45, 111–120. 14960775

[ref45] Luo Z. Yang Q. Geng B. Jiang S. Yang S. Li X. (2018). Whole genome engineering by synthesis. Sci. China Life Sci. 61, 1515–1527. 10.1007/s11427-018-9403-y 30465231

[ref46] Malik D. J. Sokolov I. J. Vinner G. K. Mancuso F. Cinquerrui S. Vladisavljevic G. T.. (2017). Formulation, stabilisation and encapsulation of bacteriophage for phage therapy. Adv. Colloid Interf. Sci. 249, 100–133. 10.1016/j.cis.2017.05.014, PMID: 28688779

[ref47] Mancuso F. Shi J. Malik D. J. (2018). High throughput manufacturing of bacteriophages using continuous stirred tank bioreactors connected in series to ensure optimum host bacteria physiology for phage production. Viruses 10:1. 10.3390/v10100537, PMID: 30275405 PMC6213498

[ref48] McCallin S. Alam Sarker S. Barretto C. Sultana S. Berger B. Huq S. (2013). Safety analysis of a Russian phage cocktail: from MetaGenomic analysis to oral application in healthy human subjects. Virology 443, 187–196. 10.1016/J.VIROL.2013.05.022 23755967

[ref49] Merabishvili M. Monserez R. van Belleghem J. Rose T. Jennes S. De Vos D.. (2017). Stability of bacteriophages in burn wound care products. PLoS One 12:e0182121. 10.1371/journal.pone.0182121, PMID: 28750102 PMC5531522

[ref50] Merabishvili M. Pirnay J. P. Verbeken G. Chanishvili N. Tediashvili M. Lashkhi N.. (2009). Quality-controlled small-scale production of a well-defined bacteriophage cocktail for use in human clinical trials. PLoS One 4:e4944. 10.1371/journal.pone.0004944, PMID: 19300511 PMC2654153

[ref51] Merten O.-W. Gény-Fiamma C. Douar A. M. (2005). Current issues in adeno-associated viral vector production. Gene Ther. 12, S51–S61. 10.1038/sj.gt.3302615, PMID: 16231056

[ref52] Metzger J. F. Johnson A. D. Collins W. S. McGann V. (1973). *Staphylococcus aureus* enterotoxin B release (excretion) under controlled conditions of fermentation. Appl. Microbiol. 25, 770–773. Available at: http://www.ncbi.nlm.nih.gov/pubmed/4715555 4715555 10.1128/am.25.5.770-773.1973 PMC380909

[ref53] Mizoguchi K. Morita M. Fischer C. R. Yoichi M. Tanji Y. Unno H. (2003). Coevolution of bacteriophage PP01 and *Escherichia coli* O157:H7 in continuous culture. Appl. Environ. Microbiol. 69, 170–176. 10.1128/AEM.69.1.170-176.2003, PMID: 12513992 PMC152390

[ref55] Neverov A. Chumakov K. (2010). Massively parallel sequencing for monitoring genetic consistency and quality control of live viral vaccines. Proc. Natl. Acad. Sci. U. S. A. 107, 20063–20068. 10.1073/pnas.1012537107 21041640 PMC2993378

[ref56] Otto M. (2014). *Staphylococcus aureus* toxins. Curr. Opin. Microbiol. 17, 32–37. 10.1016/j.mib.2013.11.004, PMID: 24581690 PMC3942668

[ref57] Petsch D. (2000). Endotoxin removal from protein solutions. J. Biotechnol. 76, 97–119. 10.1016/S0168-1656(99)00185-6, PMID: 10656326

[ref58] Pirnay J.-P. Blasdel B. G. Bretaudeau L. Buckling A. Chanishvili N. Clark J. R.. (2015). Quality and safety requirements for sustainable phage therapy products. Pharm. Res. 32, 2173–2179. 10.1007/s11095-014-1617-7, PMID: 25585954 PMC4452253

[ref59] Pirnay J. Merabishvili M. Van Raemdonck H. De Vos D. Verbeken G. (2018b). “Bacteriophage production in compliance with regulatory requirements” in Bacteriophage therapy. eds. Azeredo J. Sillankorva S. (New York, NY: Humana Press), 233–252. 10.1007/978-1-4939-7395-8_18 29119444

[ref60] Pirnay J.-P. Verbeken G. Ceyssens P.-J. Huys I. De Vos D. Ameloot C. (2018a). The magistral phage. Viruses 10:64. 10.3390/v10020064 PMC5850371 29415431

[ref61] Rajnovic D. Muñoz-Berbel X. Mas J. (2019). Fast phage detection and quantification: an optical density-based approach. PLoS One 14:e0216292. 10.1371/journal.pone.0216292, PMID: 31071103 PMC6508699

[ref62] Rhoads D. D. Wolcott R. D. Kuskowski M. A. Wolcott B. M. Ward L. S. Sulakvelidze A. (2014). Bacteriophage therapy of venous leg ulcers in humans: results of a phase I safety trial. J. Wound Care 18, 237–243. 10.12968/jowc.2009.18.6.42801 19661847

[ref63] Rodrigues G. A. Shalaev E. Karami T. K. Cunningham J. Slater N. K. H. Rivers H. M. (2019). Pharmaceutical development of AAV-based gene therapy products for the eye. Pharm. Res. 36:29. 10.1007/s11095-018-2554-7 PMC6308217 30591984

[ref64] Rose T. Verbeken G. De Vos D. Merabishvili M. Vaneechoutte M. Lavigne R. (2014). Experimental phage therapy of burn wound infection: difficult first steps. Int. J. Burn. Trauma 4, 66–73. https://www.ncbi.nlm.nih.gov/pmc/articles/PMC4212884/PMC4212884 25356373

[ref65] Rouse J. C. Yu C. Richardson D. D. Abernathy M. Rogers R. S. Swann P. (2017). A view on the importance of “multi-attribute method” for measuring purity of biopharmaceuticals and improving overall control strategy. AAPS J. 20:7. 10.1208/s12248-017-0168-3 29192343

[ref66] Samson J. E. Moineau S. (2013). Bacteriophages in food fermentations: new Frontiers in a continuous arms race. Annu. Rev. Food Sci. Technol. 4, 347–368. 10.1146/annurev-food-030212-182541, PMID: 23244395

[ref67] Sarker S. A. Sultana S. Reuteler G. Moine D. Descombes P. Charton F.. (2016). Oral phage therapy of acute bacterial diarrhea with two coliphage preparations: a randomized trial in children from Bangladesh. EBioMedicine 4, 124–137. 10.1016/j.ebiom.2015.12.023, PMID: 26981577 PMC4776075

[ref68] Sauvageau D. Cooper D. G. (2010). Two-stage, self-cycling process for the production of bacteriophages. Microb. Cell Factories 9:81. 10.1186/1475-2859-9-81, PMID: 21040541 PMC2989940

[ref69] Schmitt S. (Ed.) (2011). Quality by design: Putting theory into practice. MD, USA: Davis Healthcare International Publishing.

[ref70] Schwarz H. Gornicec J. Neuper T. Parigiani M. A. Wallner M. Duschl A.. (2017). Biological activity of masked endotoxin. Sci. Rep. 7:44750. 10.1038/srep44750, PMID: 28317862 PMC5357793

[ref71] Shintani H. (2016). Validation study of rapid assays of bioburden, endotoxins and other contamination. Biocontrol Sci. 21, 63–72. 10.4265/bio.21.63, PMID: 27350424

[ref72] Souvignier C. Mamat U. Corchero J. L. Schromm A. B. Schaffer L. Meredith T. C.. (2015). Detoxifying *Escherichia coli* for endotoxin-free production of recombinant proteins. Microb. Cell Factories 14:57. 10.1186/s12934-015-0241-5, PMID: 25890161 PMC4404585

[ref520] Storms Z. J. Teel M. R. Mercurio K. Sauvageau D. (2019). The virulence index: a metric for quantitative analysis of phage virulence. Phage 1, 17–26. 10.1089/phage.2019.0001 PMC9041455 36147620

[ref73] Sturino J. M. Klaenhammer T. R. (2006). Engineered bacteriophage-defence systems in bioprocessing. Nat. Rev. Microbiol. 4, 395–404. 10.1038/nrmicro1393, PMID: 16715051

[ref74] Sweere J. M. Van Belleghem J. D. Ishak H. Bach M. S. Popescu M. Sunkari V.. (2019). Bacteriophage trigger anti-viral immunity and prevent clearance of bacterial infection. Science 363:eaat9691. 10.1126/science.aat9691, PMID: 30923196 PMC6656896

[ref75] Szermer-Olearnik B. Boratyński J. (2015). Removal of endotoxins from bacteriophage preparations by extraction with organic solvents. PLoS One 10:e0122672. 10.1371/journal.pone.0122672, PMID: 25811193 PMC4374689

[ref76] Van Belleghem J. D. Merabishvili M. Vergauwen B. Lavigne R. Vaneechoutte M. (2017). A comparative study of different strategies for removal of endotoxins from bacteriophage preparations. J. Microbiol. Methods 132, 153–159. 10.1016/j.mimet.2016.11.020 27913133

[ref77] Villarroel J. Larsen M. Kilstrup M. Nielsen M. Villarroel J. Larsen M. V.. (2017). Metagenomic analysis of therapeutic PYO phage cocktails from 1997 to 2014. Viruses 9:328. 10.3390/v9110328, PMID: 29099783 PMC5707535

[ref78] WHO Technical Report Series No. 926 (2018). Guidelines for the Safe Production and Quality Control of Poliomyelitis Vaccine Revision of Annex 2 of WHO Technical Report Series, No. 926. Available at: https://www.who.int/biologicals/expert_committee/POST_ECBS_2018_Polio_Web_9_Nov_2018.pdf?ua=1 (Accessed April 10, 2019).

[ref79] Wright A. Hawkins C. H. Änggård E. E. Harper D. R. (2009). A controlled clinical trial of a therapeutic bacteriophage preparation in chronic otitis due to antibiotic-resistant *Pseudomonas aeruginosa*; a preliminary report of efficacy. Clin. Otolaryngol. 34, 349–357. 10.1111/j.1749-4486.2009.01973.x, PMID: 19673983

[ref80] Xie Y. Wahab L. Gill J. Xie Y. Wahab L. Gill J. J. (2018). Development and validation of a microtiter plate-based assay for determination of bacteriophage host range and virulence. Viruses 10:189. 10.3390/v10040189, PMID: 29649135 PMC5923483

[ref81] Yu L. X. Amidon G. Khan M. A. Hoag S. W. Polli J. Raju G. K.. (2014). Understanding pharmaceutical quality by design. AAPS J. 16, 771–783. 10.1208/s12248-014-9598-3, PMID: 24854893 PMC4070262

[ref82] Yuan Y. Peng Q. Zhang S. Liu T. Yang S. Yu Q.. (2019). Phage reduce stability for regaining infectivity during antagonistic coevolution with host bacterium. Viruses 11:118. 10.3390/v11020118, PMID: 30699954 PMC6410104

[ref83] Zhang R. Li C. Wang J. Yang Y. Yan Y. (2018). Microbial production of small medicinal molecules and biologics: from nature to synthetic pathways. Biotechnol. Adv. 36, 2219–2231. 10.1016/j.biotechadv.2018.10.009, PMID: 30385278

